# Dopamine and the Brainstem Locomotor Networks: From Lamprey to Human

**DOI:** 10.3389/fnins.2017.00295

**Published:** 2017-05-26

**Authors:** Dimitri Ryczko, Réjean Dubuc

**Affiliations:** ^1^Groupe de Recherche sur le Système Nerveux Central, Département de Neurosciences, Université de MontréalMontréal, QC, Canada; ^2^Groupe de Recherche en Activité Physique Adaptée, Département des Sciences de l'Activité Physique, Université du Québec à MontréalMontréal, QC, Canada

**Keywords:** locomotion, brainstem, dopamine, mesencephalic locomotor region, *substantia nigra pars compacta*, pedunculopontine nucleus, conservation, Parkinson's disease

## Abstract

In vertebrates, dopamine neurons are classically known to modulate locomotion via their ascending projections to the basal ganglia that project to brainstem locomotor networks. An increased dopaminergic tone is associated with increase in locomotor activity. In pathological conditions where dopamine cells are lost, such as in Parkinson's disease, locomotor deficits are traditionally associated with the reduced ascending dopaminergic input to the basal ganglia. However, a descending dopaminergic pathway originating from the *substantia nigra pars compacta* was recently discovered. It innervates the mesencephalic locomotor region (MLR) from basal vertebrates to mammals. This pathway was shown to increase locomotor output in lampreys, and could very well play an important role in mammals. Here, we provide a detailed account on the newly found dopaminergic pathway in lamprey, salamander, rat, monkey, and human. In lampreys and salamanders, dopamine release in the MLR is associated with the activation of reticulospinal neurons that carry the locomotor command to the spinal cord. Dopamine release in the MLR potentiates locomotor movements through a D1-receptor mechanism in lampreys. In rats, stimulation of the *substantia nigra pars compacta* elicited dopamine release in the pedunculopontine nucleus, a known part of the MLR. In a monkey model of Parkinson's disease, a reduced dopaminergic innervation of the brainstem locomotor networks was reported. Dopaminergic fibers are also present in human pedunculopontine nucleus. We discuss the conserved locomotor role of this pathway from lamprey to mammals, and the hypothesis that this pathway could play a role in the locomotor deficits reported in Parkinson's disease.

## Ascending dopaminergic pathway and locomotion

Dopaminergic neurons degenerate in patients with Parkinson's disease (PD), resulting in serious motor dysfunctions including locomotor deficits (falls, gait freezing, dysfunctional turning), which constitute major problems in advanced forms of the disease (Stack and Ashburn, 2008, for review see Bloem et al., 2004). These locomotor deficits are traditionally associated with a loss of the ascending dopaminergic projections from the *substantia nigra pars compacta* (SNc) to the basal ganglia (Carlsson et al., 1958; Carlsson, 1959; Sano et al., 1959; Poirier and Sourkes, 1965; Sourkes and Poirier, 1965; Albin et al., 1989; Ehringer and Hornykiewicz, 1998; Kravitz et al., 2010; Roseberry et al., 2016, for review see Fahn, 2015). In turn, the basal ganglia project down to the Mesencephalic Locomotor Region (MLR), a brainstem region that controls locomotion in vertebrates (Shik et al., 1966; for review see Ryczko and Dubuc, 2013, Figure 1). The MLR was initially found in cats to initiate locomotion and control its frequency and intensity (Shik et al., 1966). It was later identified in lamprey (Sirota et al., 2000), salamander (Cabelguen et al., 2003), stingray (Bernau et al., 1991), bird (Sholomenko et al., 1991), rat (Garcia-Rill et al., 1987), mouse (Lee et al., 2014; Roseberry et al., 2016), rabbit (Musienko et al., 2008), guinea-pig (Marlinsky and Voitenko, 1991), and monkey (Eidelberg et al., 1981; Karachi et al., 2010; Goetz et al., 2016a). In basal vertebrates, the MLR comprises the laterodorsal tegmental nucleus and the pedunculopontine nucleus (PPN). In mammals, it comprises the PPN, but also the cuneiform nucleus (CnF). In humans, damage to the MLR is associated with locomotor deficits (Masdeu et al., 1994; Kuo et al., 2008; Demain et al., 2014). The MLR is explored as a target for deep brain stimulation to improve locomotor function in Parkinsonian patients (Plaha and Gill, 2005; for review see Hamani et al., 2016a,b).

**Figure 1:**
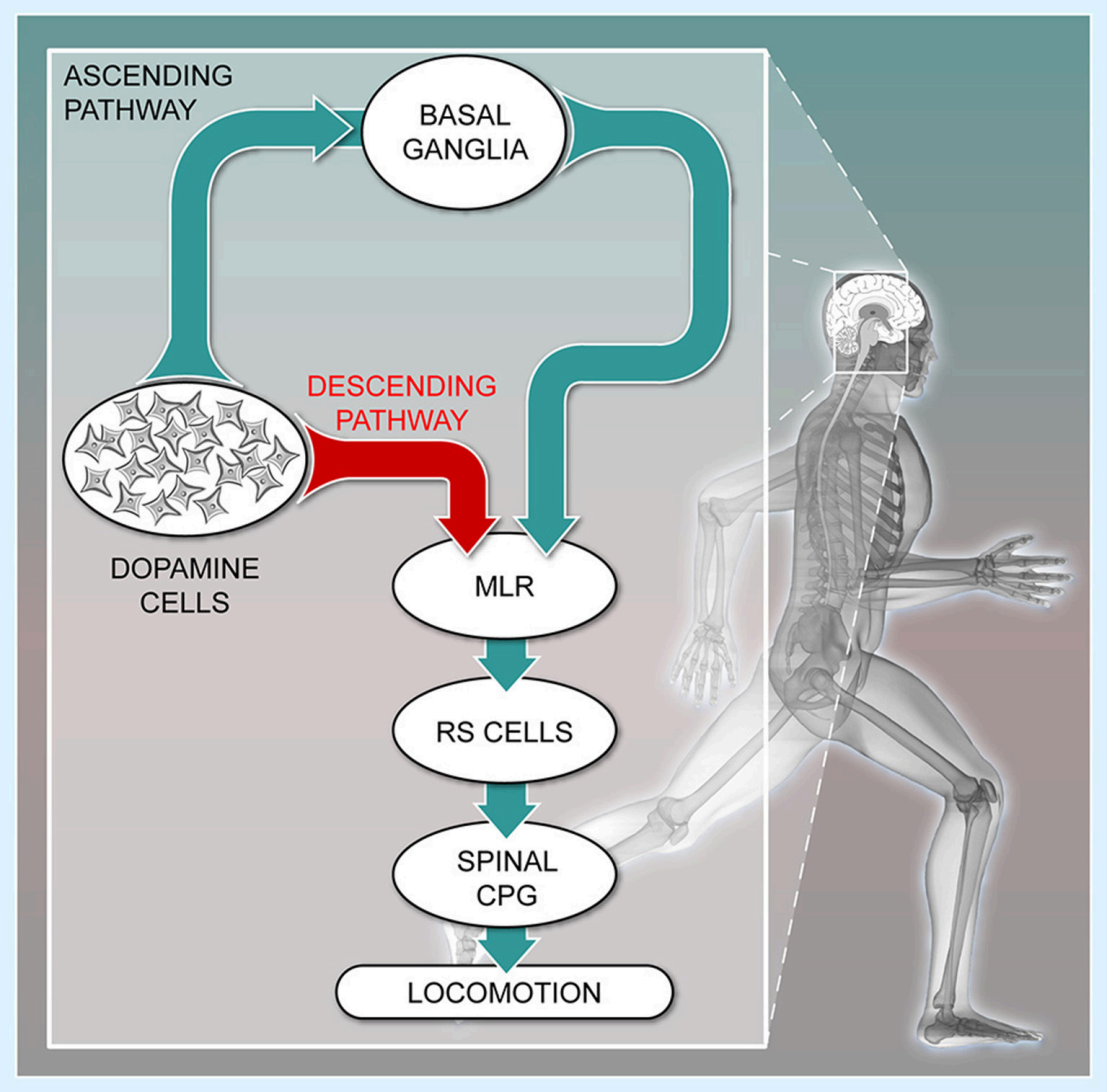
**The descending dopaminergic pathway recently uncovered in vertebrates**. Schematic representation of the connectivity between the meso-diencephalic dopamine cells, the basal ganglia, the Mesencephalic Locomotor Region (MLR), the reticulospinal cells (RS), and the Central Pattern Generator (CPG) for locomotion. The meso-diencephalic dopamine cells refer to the posterior tuberculum in basal vertebrates and to the *substantia nigra pars compacta* in mammals. For convenience, the well-established direct and indirect pathways within the basal ganglia are not illustrated. (Adapted from (Le Ray et al., 2011). No permission is required for this reproduction).

The ascending dopaminergic projections mostly target the striatum, a major entry of the basal ganglia. These projections favor locomotion initiation by increasing the excitability of D_1_-expressing striatal neurons of the direct pathway, and this reduces the tonic inhibition sent by the output stations of the basal ganglia to the MLR. In parallel, dopamine decreases the excitability of D_2_-expressing striatal neurons of the indirect pathway. This also contributes to disinhibit the MLR, and initiate movement (Albin et al., 1989; Kravitz et al., 2010; Freeze et al., 2013; Roseberry et al., 2016). Such organization is conserved within the basal ganglia from lamprey to mammals (see Grillner and Robertson, 2016). Once disinhibited, the MLR initiates locomotion by sending descending excitatory inputs to reticulospinal neurons, which activate the central pattern generator for locomotion (Figure 1, cat: Orlovskii, 1970; Steeves and Jordan, 1980; Garcia-Rill and Skinner, 1987a,b; Noga et al., 1988, 1991; rat: Bachmann et al., 2013; bird: Sholomenko et al., 1991; lamprey: Buchanan and Grillner, 1987; Brodin et al., 1988; Ohta and Grillner, 1989; Brocard and Dubuc, 2003; Le Ray et al., 2003; mouse: Bretzner and Brownstone, 2013; salamander: Ryczko et al., 2016b). MLR glutamatergic neurons are of primary importance to activate reticulospinal neurons and elicit locomotion (lamprey: Brocard and Dubuc, 2003, salamander: Ryczko et al., 2016b, mouse: Lee et al., 2014; Roseberry et al., 2016). MLR cholinergic neurons provide additional excitation to reticulospinal cells (lamprey: Le Ray et al., 2003; Smetana et al., 2010; mouse: Roseberry et al., 2016). The functional significance of this circuitry was elegantly summed in a mouse study, in which it was shown that ascending dopaminergic pathways to the basal ganglia indirectly control MLR glutamatergic cells and locomotion (Roseberry et al., 2016). The loss of the ascending dopaminergic pathway is thus considered the main cause of locomotor deficits in PD.

## A new descending dopaminergic pathway has been unraveled

There was some indication in the literature that in addition to their ascending projections, dopaminergic cells also sent direct descending projections to brainstem locomotor networks. In rat, dopamine was detected using radiometric assays or microdialysis in the CnF (Versteeg et al., 1976; Saavedra et al., 1979) and PPN (Steiniger and Kretschmer, 2003) that are both part of the MLR in mammals (see Ryczko and Dubuc, 2013). Moreover, dopaminergic fibers were detected in rat brainstem using immunohistochemistry (Kitahama et al., 2000). In monkey, dopaminergic terminals were found in proximity with cholinergic cells of the PPN and CnF (Rolland et al., 2009). The origin of this dopaminergic projection remained unknown, but tracing studies mentioned a descending projection from the SNc to the PPN in rat (Beckstead et al., 1979; Semba and Fibiger, 1992; Steininger et al., 1992; Ichinohe et al., 2000) and in cat (Edley and Graybiel, 1983). The presence of such descending input was also supported by recordings of short latency antidromic activation of SNc neurons following PPN stimulation in rat (Scarnati et al., 1984, 1987).

**Figure 2:**
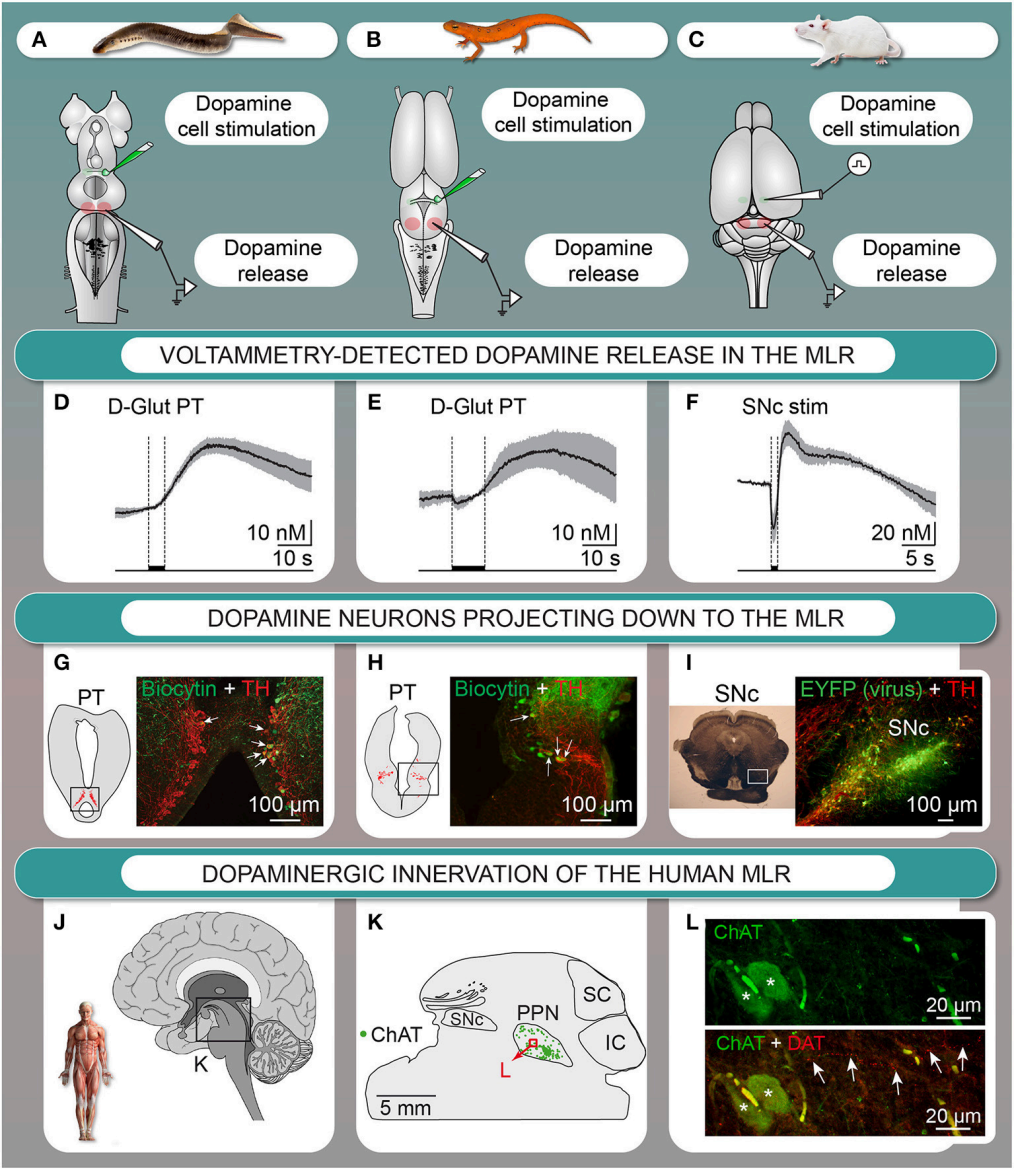
**The descending dopaminergic (DA) pathway is conserved from basal vertebrates to mammals. (A–F)** Dopamine is released in the MLR after chemical stimulation of DA cells in the posterior tuberculum (PT) of lampreys **(A,D)** or salamanders (*in vitro* isolated brain) **(B,E)**, or after electrical stimulation of the *substantia nigra pars compacta* (SNc) in rats (anesthetized) **(C,F)**. **(D–F)** mean ± sem is illustrated. **(G)** Lamprey tyrosine hydroxylase (TH)-containing cells (red) in the PT and cells projecting to the MLR (green) with arrows indicating double labeled cells. **(H)** Salamander TH-containing cells (red) in the PT and cells projecting to the MLR (green) with arrows indicating double labeled cells. **(I)** DA cells in the SNc retrogradely labeled by an injection of a Cre-dependent adeno-associated virus encoding for the enhanced yellow fluorescent protein (EYFP, green) in the MLR of transgenic rats expressing the Cre-recombinase in TH neurons as shown by immunostaining against TH (red). **(J–L)** DA innervation of the human MLR. **(J–L)** The location of cholinergic cells (choline acetyltransferase-positive, ChAT) of the pedunculopontine nucleus (PPN), part of the MLR, is indicated. **(L)** Fibers containing the dopamine active transporter (DAT, red, highlighted by arrows) in proximity with cholinergic cells (ChAT, green) in the PPN. IC, inferior colliculus; SC, superior colliculus. (Panels **A,D,G** adapted from D. Ryczko, S. Gratsch, F. Auclair, C. Dube, S. Bergeron, M.H. Alpert, J.J. Cone, M.F. Roitman, S. Alford, and R. Dubuc, Forebrain dopamine neurons project down to a brainstem region controlling locomotion. Proceedings of the National Academy of Sciences of the United States of America 110 (2013) E3235–E3242. No permission is required for this reproduction; panels **B,C,E,F,H,I,J–L** adapted from D. Ryczko, J.J. Cone, M.H. Alpert, L. Goetz, F. Auclair, C. Dube, M. Parent, M.F. Roitman, S. Alford, and R. Dubuc, A descending dopamine pathway conserved from basal vertebrates to mammals. Proceedings of the National Academy of Sciences of the United States of America 113 (2016) E2440–E2449. No permission is required for this reproduction).

We investigated the origin of the dopaminergic innervation of the MLR in vertebrates. In lamprey (Ryczko et al., 2013) and salamander (Ryczko et al., 2016a), dopaminergic fibers were found around MLR cholinergic cells, a conserved landmark of the MLR (see Ryczko and Dubuc, 2013). We identified the origin of this dopaminergic innervation in lamprey (Figure 2G, Ryczko et al., 2013; see also Perez-Fernandez et al., 2014) and in salamander (Figure 2H, Ryczko et al., 2016a) as a diencephalic dopaminergic region called the posterior tuberculum. This region sends ascending projection to the striatum, and is considered homologous to the mammalian SNc and/or ventral tegmental area (Marin et al., 1997; Pombal et al., 1997; Puelles and Verney, 1998; Smeets et al., 2000; Rink and Wullimann, 2001; Blin et al., 2008; for review see Yamamoto and Vernier, 2011; Wullimann, 2014). We then found that such “new pathway” (Figure 1) is conserved in higher vertebrates. In rat, PPN cholinergic cells were innervated by dopaminergic fibers (Ryczko et al., 2016a). Using virogenetic tracing, we found that the dopaminergic innervation of the rat MLR originates from the SNc and to a lesser extent the retrorubral field (Figure 2I, Ryczko et al., 2016a). This was confirmed using conventional tracers coupled with immunofluorescence experiments (Ryczko et al., 2016a). While only a few dopamine neurons sent both an ascending projection to the striatum and a descending one to the MLR in lampreys and salamanders, numerous SNc dopamine neurons sent both ascending and descending projections in rats. The proportion of the ascending dopaminergic projection may have increased during evolution due to the expansion of the basal ganglia (see Grillner and Robertson, 2016). We then found in the human brain that PPN cholinergic cells are surrounded by dopaminergic fibers (Figures 2J–L, Ryczko et al., 2016a), indicating that the innervation of the MLR is conserved in vertebrates.

The descending dopaminergic pathway was shown to release dopamine in the MLR with fast-scan voltammetry (Ryczko et al., 2016a). Stimulation of the dopaminergic region evoked dopamine release in the MLR *in vitro* in lamprey (Figures 2A,D, Ryczko et al., 2013) and in salamander (Figures 2B,E, Ryczko et al., 2016a). In rat, SNc stimulation evoked dopamine release in the PPN *in vivo* (Figures 2C,F) that was potentiated by intraperitoneal amphetamine injection (Ryczko et al., 2016a). Altogether, these results established that the descending dopaminergic pathway is conserved and functional from basal vertebrates (lampreys, salamanders) to mammals (rats).

The role of the descending dopaminergic pathway in modulating locomotor activity was examined in two basal vertebrates. In lampreys and salamanders, stimulation of the dopamine region evoked dopamine release in the MLR, associated with activation of reticulospinal cells, which carry the locomotor command to the spinal cord (Ryczko et al., 2013, 2016a). There was a precise correlation in time linking MLR dopamine release and the activation of reticulospinal cells. The behavioral role of dopamine release in the MLR was examined in a lamprey semi-intact preparation (Ryczko et al., 2013), where the brain is exposed while the body swims as reported in many studies from our group (Sirota et al., 2000; Viana Di Prisco et al., 2000; Le Ray et al., 2003; Brocard et al., 2005, 2010; Gravel et al., 2007; Menard et al., 2007; Derjean et al., 2010; Smetana et al., 2010; Gariepy et al., 2012; Juvin et al., 2016). Stimulation of the dopaminergic region elicited reticulospinal activity together with locomotion, and microinjections of a D_1_ antagonist in the MLR decreased the number of locomotor cycles, the frequency of locomotor movements, and the duration of the locomotor bout (Ryczko et al., 2013). Conversely, microinjection of dopamine in the MLR had an opposite effect (Ryczko et al., 2013). In mammals, whether MLR dopamine release is associated with activation of the locomotor system remains to be addressed. The observation that amphetamine increases dopamine release in the rat MLR (Ryczko et al., 2016a) suggests an involvement of the descending dopaminergic pathway in the well-characterized increase in locomotor activity elicited by dopaminergic drugs (e.g., psychostimulants, L-DOPA).

The mechanisms through which dopamine potentiates MLR cell activity remain to be determined. It is possible that MLR dopamine enhances locomotor output by potentiating glutamatergic inputs to the MLR. In support of this, stimulation of the dopaminergic region evokes fast excitatory synaptic inputs in MLR cells in lampreys (Gariepy et al., 2012; Ryczko et al., 2013). This fast input could be glutamatergic and monosynaptic according to anatomical and electrophysiological data (Derjean et al., 2010). Future research should determine whether the two transmitters cooperate pre- and/or post-synaptically, and establish the role of dopaminergic inputs on intrinsic properties of MLR cells.

## Possible role of the descending dopaminergic pathway in PD

There is accumulating evidence indicating that the MLR plays a similar role in humans as described in animal models. Moreover, it appears that some of the locomotor deficits observed in PD can be attributed to changes in the brainstem locomotor circuitry including the MLR. The PPN and CnF, both parts of the MLR, are activated in healthy individuals when they are asked to imagine that they are walking (Jahn et al., 2008; Snijders et al., 2011; Karachi et al., 2012; Peterson et al., 2014; Tattersall et al., 2014). In Parkinsonian subjects, similar observations were reported (Piallat et al., 2009; Lau et al., 2015; for review see Bohnen and Jahn, 2013). PPN activity increases during walking, and is modulated by L-DOPA with increase in alpha band (5–12 Hz) and decrease in beta (13–35) and gamma (65–90 Hz) bands (Fraix et al., 2013). Gait freezing is associated with a decreased alpha band activity in the PPN (Thevathasan et al., 2012). Motor arrests are associated with decreased blood oxygen levels in the MLR (Shine et al., 2013). Neuronal losses were reported in the PPN of patients with PD or progressive supranuclear palsy (Hirsch et al., 1987; Zweig et al., 1987, 1989; Jellinger, 1988). In PD this includes degeneration of cholinergic (Rinne et al., 2008; Karachi et al., 2010; Pienaar et al., 2013), GABAergic and glycinergic cells (Pienaar et al., 2013). Neuroimaging indicates that locomotor deficits in PD patients are associated with additional MLR abnormalities (notably in the PPN), including altered connectivity between the MLR, thalamus, and motor cortical regions (Fling et al., 2013, 2014), abnormal microstructure (Vercruysse et al., 2015; Youn et al., 2015; Wang et al., 2016), atrophy of the MLR gray matter (Snijders et al., 2011; Fioravanti et al., 2015) and abnormal metabolic activity following a walking task (Tard et al., 2015). Additionally, anatomopathological studies revealed the presence in the MLR of alpha-synuclein immuno-reactive Lewy Bodies (e.g., Seidel et al., 2015), and mitochondrial abnormalities (Pienaar et al., 2013) in PD. The severity of the locomotor deficits increases with the amplitude of PPN damage as captured by neuroimaging (Canu et al., 2015). These data are consistent with those showing that non-Parkinsonian individuals with MLR lesion display locomotor deficits (Masdeu et al., 1994; Kuo et al., 2008; Yeo et al., 2012), and that elderly with high level gait and balance disorders display midbrain gray matter atrophy including in the MLR (Demain et al., 2014). Finally, more and more studies point to the involvement of the PPN in the locomotor improvements related to deep brain stimulation of the subthalamic nucleus (human: Holiga et al., 2015; Knight et al., 2015; Weiss et al., 2015), which sends excitatory glutamatergic input to the PPN (e.g., Breit et al., 2001; Neagu et al., 2013; see Ryczko and Dubuc, 2013).

The benefits of MLR deep brain stimulation on locomotor function in PD (Plaha and Gill, 2005) are variable, from promising to modest (for recent studies, see Schrader et al., 2013; Mazzone et al., 2014; Holiga et al., 2015; Liu et al., 2015; Nosko et al., 2015; Welter et al., 2015) or unsustained benefits over the years (Mestre et al., 2016). This variability could be attributed to degeneration of MLR cells and to the variability of the brainstem anatomy from patient to patient (Mazzone et al., 2013). Reviewing the fast-growing body of literature on this neurosurgical approach is beyond the scope of the present review (for recent reviews, see Collomb-Clerc and Welter, 2015; DeLong and Wichmann, 2015; Fasano et al., 2015; Golestanirad et al., 2016; Rowe et al., 2016; Snijders et al., 2016). Several authors pointed out that adequate control trials and more standardization are needed before concluding on the efficacy of MLR deep brain stimulation (Windels et al., 2015; for review, see Hamani et al., 2016a,b).

The dopaminergic innervation of the PPN and CnF dramatically degenerates in a monkey model of PD (Rolland et al., 2009). The degeneration elicited by MPTP was even more marked in aged monkeys, maybe underlining the increasing fragility of this innervation over lifetime. The loss of dopaminergic innervation in the MLR could contribute to the pathophysiology of PD in several ways. If the role of the descending dopaminergic pathway to the MLR is conserved in higher vertebrates, locomotor deficits in PD may result, at least in part, from the loss of excitatory dopaminergic inputs to the MLR. This would lead to a reduced amplification of descending locomotor commands. Conversely, the descending dopaminergic pathway may improve locomotor function evoked by L-DOPA in people with PD (e.g., Moore et al., 2008; Chastan et al., 2009; Bryant et al., 2011a,b) by increasing the excitability of MLR cells. Importantly, locomotor deficits that are unresponsive to L-DOPA are associated with MLR degeneration (Chastan et al., 2009; Karachi et al., 2010; Snijders et al., 2011). It is thus possible that the beneficial effects of increasing dopamine release in the MLR with L-DOPA, or of stimulating MLR cells with dopaminergic agonists could improve locomotor function before MLR cells are lost in large number.

It is also possible that the loss of dopaminergic inputs to the MLR may disrupt the excitability of MLR cells, causing them to eventually degenerate. Such *transneuronal degeneration* can occur anterogradely or retrogradely and is characterized by a “structural deterioration of areas remote from the initial insult” (Fornito et al., 2015). This phenomenon was shown in the visual (e.g., Hubel and Wiesel, 1970; Herbin et al., 1999) and olfactory systems (e.g., Pinching and Powell, 1971). Transneuronal degeneration was also shown to damage dopaminergic neurons following striatal lesion (Macaya et al., 1994; Marti et al., 1997; El-Khodor and Burke, 2002; Canudas et al., 2005) and was proposed to contribute to PD (Pedersen and Schmidt, 2000). It was also proposed to occur in other neurodegenerative diseases including Alzheimer's disease and amyotrophic lateral sclerosis (see Fornito et al., 2015). The multiple alterations in the MLR in PD are compatible with such phenomenon (see Fornito et al., 2015). The reciprocal projections between the SNc and the PPN (McGeer and McGeer, 1984; Lavoie and Parent, 1994; Ryczko et al., 2013, 2016a; Perez-Fernandez et al., 2014) could also contribute to potentiate the transneuronal degeneration process. Nigral dopamine cell degeneration would cause a loss of the dopaminergic input to the MLR, causing MLR cells to degenerate. In turn, degeneration of PPN cholinergic and glutamatergic cells projecting to the nigral dopamine neurons would contribute to nigral dopamine cell loss. Studies in rat and monkey indicate that destruction of dopamine cells causes degeneration of MLR cholinergic cells (Pienaar et al., 2015b; Bensaid et al., 2016). Conversely, lesion of PPN cholinergic neurons induces a loss of dopaminergic nigral neurons (Bensaid et al., 2016). Finally, lesion of nigral dopaminergic neurons followed by lesion of PPN cholinergic cells induces a more dramatic degeneration of PPN cholinergic cells (Bensaid et al., 2016), suggesting that the two lesions interact to create a transneuronal degeneration loop. Stabilization of the reciprocal interactions between dopamine and cholinergic neurons could be a promising avenue to alleviate degeneration of the two systems. Interestingly, activation of PPN cholinergic cells with designer receptors exclusively activated by designer drugs (DREADDs) improves locomotor function in a rat model of PD (Pienaar et al., 2015a). It would be interesting to determine whether this approach would decrease degeneration of cholinergic and dopaminergic cells.

The descending dopaminergic projections to the PPN could also regulate other important functions such as cognition, sleep (Stefani et al., 2013; Karachi and Francois, 2017), modulation of visual inputs during locomotion (Lee et al., 2014), arousal state (Garcia-Rill et al., 2015a,b; Goetz et al., 2016b), motivation, and reward (Xiao et al., 2016; Yoo et al., 2017). How the descending dopaminergic input to the PPN influences these functions should be the subject of future research. Interestingly, the multifunctional aspects of the MLR, well established in lampreys (i.e., regulation of locomotion, respiration, control of sensory inputs, see Ryczko and Dubuc, 2013), are mirrored by the multifunctionality of the PPN in mammals.

In conclusion, studies carried out in two basal vertebrates (lampreys and salamanders) allowed us to discover a direct dopaminergic projection from the SNc down to the MLR. Several lines of evidence indicate that this new dopaminergic pathway is functional in rats, and could also be present in humans. Future research should address whether the descending dopaminergic pathway potentiates locomotion in mammals as in basal vertebrates, whether it contributes to other PPN functions, and whether this dopaminergic innervation degenerates in PD patients.

## Author contributors

DR and RD wrote the article.

### Conflict of interest statement

The authors declare that the research was conducted in the absence of any commercial or financial relationships that could be construed as a potential conflict of interest.
